# Sympathetic overactivity prevails over the vascular amplifier phenomena in a chronic kidney disease rat model of hypertension

**DOI:** 10.14814/phy2.12205

**Published:** 2014-11-20

**Authors:** Omar Z. Ameer, Cara M. Hildreth, Jacqueline K. Phillips

**Affiliations:** 1 The Australian School of Advanced Medicine, Macquarie University, Sydney, New South Wales, Australia

**Keywords:** Chronic kidney disease, ganglion blockade, sympathetic nervous system, vascular amplifier

## Abstract

We examined whether increased sympathetic nerve activity (SNA) accounts for enhanced depressor responses to ganglionic blockade in the Lewis polycystic kidney (LPK) model of chronic kidney disease (CKD) or whether it reflects increased vascular responses to vasodilation (vascular amplifier). Under urethane anesthesia, depressor responses to ganglionic blockade (hexamethonium, 0.5–40 mg/kg i.v.), and direct vasodilation (sodium nitroprusside [SNP], 2.5–40 *μ*g/kg i.v. and adenosine, 3–300 *μ*g/kg i.v.) were compared in the LPK with normotensive Lewis and spontaneously hypertensive rats (SHR) (total *n* = 37). Hexamethonium (8 mg/kg) produced a greater depressor response in the LPK (−51 ± 3 mmHg) compared with Lewis (−31 ± 3 mmHg, *P *<**0.05) but not SHR (−46 ± 3 mmHg). In LPK, the ratio of the hexamethonium/vasodilator MAP responses was greater when compared with Lewis (hexamethonium/SNP 1.34 ± 0.1 vs. 0.9 ± 0.09 and hexamethonium/adenosine: 2.28 ± 0.3 vs. 1.16 ± 0.1, both *P *<**0.05) but not SHR. Results for systolic blood pressure (SBP) were comparable. The slope of the relationship between the fall in SBP induced by hexamethonium and normalized low frequency (LF_nu_) power was also greater in the LPK (17.93 ± 3.26 mmHg/LF_nu_) compared with Lewis (2.78 ± 0.59 mmHg/LF_nu_, *P *=**0.001) and SHR (3.36 ±0.72 mmHg/LF_nu_, *P *=**0.003). These results indicate that in the LPK, sympathetic activity predominates over any vascular amplifier effect, supporting increased sympathetic vasomotor tone as a major contributor to hypertension in this model of CKD.

## Abstract (precis)

We investigated whether enhanced depressor responses to the ganglionic blocker hexamethonium is due to sympathetic overactivity or exaggerated vascular responses to vasodilation (vascular amplifier) in the Lewis polycystic kidney (LPK) rodent model of chronic kidney disease (CKD) compared to Lewis and spontaneously hypertensive rats (SHR). The main finding of our study is that sympathetic overactivity predominates over vascular amplification in response to ganglionic blockade in the LPK, indicating that increased sympathetic vasomotor tone is a major contributor to the hypertension in this model of CKD.

## Introduction

Sympathetic overactivity is well regarded as an underlying contributing cause of hypertension in patients with chronic kidney disease (CKD), with reports of both increased levels of sympathetic nerve activity (SNA) (Johansson et al. 1999; Klein et al. 2001; Grassi et al. 2011) and noradrenaline spillover (Klein et al. 2001; Zoccali et al. 2002). As a consequence of sympathetic overactivity, however, the vasculature can become hypertrophied (Bevan 1984), which in itself or as mediated by a complex interaction with the renin‐angiotensin system, can sensitize the vasculature to sympathetic vasomotor input (Adams et al. 1990; Smid et al. 1995); a phenomenon termed the *vascular amplifier effect* (Folkow and Karlstrom 1984; Adams et al. 1989; Black et al. 1997). This outcome is not specific to sympathetic input but functions to amplify any dilator or constrictor stimulus (Wright and Angus 1999). Distinguishing the contribution of the sympathetic nervous system (SNS) from a structurally‐based vascular amplifier effect to the development and/or maintenance of hypertension can therefore be problematic, because the degree of sympathetic vasomotor tone is commonly inferred by measuring the blood pressure response to withdrawal of sympathetic vasomotor input. Accordingly, a larger reduction in blood pressure in response to administration of sympatholytic agents has been argued to reflect an increased contribution of the SNS to the regulation of blood pressure (Li et al. 1996; Vanness et al. 1999; Shannon et al. 2000; Abdala et al. 2012).

Spectral analysis of blood pressure is another method used to assess the contribution of the SNS to resting blood pressure, with increases in the amount of low frequency (LF) oscillations in systolic blood pressure (SBP) suggestive of increased sympathetic vasomotor tone (Stauss 2007). However, the potential interplay between the SNS and the vasculature, and the resulting vascular amplifier effect, means that the heightened depressor responses to administration of sympatholytic agents and LF oscillations in SBP may also be inappropriately attributed to increased sympathetic vasomotor tone, as opposed to a heightened sensitivity of the vasculature to sympathetic vasomotor input. This notion was highlighted by Moretti et al. (2009) who demonstrated that the heightened depressor responses to the ganglionic blocker pentolinium in the angiotensin II‐induced hypertensive rabbit model were not in fact reflective of increased sympathetic tone, but as a consequence of a vascular amplifier effect.

Recently, we have established that the Lewis Polycystic Kidney (LPK) rat, a model of autosomal recessive cystic kidney disease due to a spontaneous mutation in the *Nek8* gene (McCooke et al. 2012), exhibits autonomic dysfunction as evidenced by increased renal SNA (Salman et al. 2014) and impaired baroreflex control of heart rate (HR) and SNA (Hildreth et al. 2013; Salman et al. 2014). The LPK rat also exhibits heightened depressor responses to administration of the ganglionic blocker hexamethonium (Phillips et al. 2007) and an increase in the LF oscillations in SBP (Harrison et al. 2010) suggesting increased sympathetic control of the vasculature in this model. However, associated with the marked hypertension exhibited in this rat model is vascular remodeling (Ng et al. 2011; Salman et al. 2014), and therefore the presumed heightened sympathetic vasomotor tone observed in this animal model may not solely reflect the increased SNA we have demonstrated (Salman et al. 2014), but also a vascular amplifier effect.

In the present study, we therefore sought to determine the relative contribution of the SNS versus the vasculature to the maintenance of blood pressure in the LPK rat. To achieve this, we measured the ratio of the depressor response to direct acting vasodilation with that of ganglionic blockade (Moretti et al. 2009) as well as the slope of the relationship between the fall in SBP induced by ganglionic blockade and the resulting level of LF power of systolic blood pressure variability (SBPV) (Diedrich et al. 2003). These measurements were made in the LPK in comparisons with two control groups: (1) The Lewis normotensive rat in which the depressor response to ganglionic blockade is mediated via sympathetic withdrawal only; and (2) the spontaneously hypertensive rat (SHR), which exhibits both increased SNA (Judy et al. 1976; Murphy et al. 1991) and a functional vascular amplifier (Adams et al. 1989, 1990).

We hypothesized that if the SNS was dominant over any vascular amplifier effect in the LPK, then the ratio of the depressor response to a direct acting vasodilator versus that of ganglionic blockade and the slope of the relationship between a fall in SBP induced by ganglionic blockade against the resulting level of LF power (Diedrich et al. 2003; Moretti et al. 2009) would be greater in the LPK rats compared with either the Lewis and SHR. If however, a vascular amplifier effect dominated then the LPK would differ from neither the Lewis nor SHR, while if a vascular amplifier effect was partially contributing to the depressor response to sympathetic withdrawal then the LPK would differ from the Lewis but not SHR.

## Methods

### Animals and ethical approval

All experiments were approved by Macquarie University Animal Ethics Committee and conducted in accordance with the Australian Code of Practice for the Care and Use of Animals for Scientific Purposes. For this study, equal numbers of mixed‐sex LPK (12–13 weeks old; *n* = 12) and normotensive Lewis rats (12–13 weeks old; *n* = 13) were used. Adult SHR aged 17–18 weeks old were also used (*n* = 12). This age of SHR was chosen as it is an age at which vascular resistance makes a greater relative contribution to the maintenance of hypertension in this strain (Adams et al. 1989).

### Surgical preparation

Rats were anesthetized with ethyl carbamate (Urethane 1.3 g/kg, i.p.; Sigma Aldrich, Castle Hill, NSW, Australia). Adequacy of anesthesia was confirmed by the lack of withdrawal to tactile (corneal stroking) and noxious (hind paw pinch) stimuli. Supplemental doses of urethane (0.13 g/kg i.v.) were administered as necessary. Body temperature was monitored using a rectal probe and maintained at 37 ± 0.5°C with a homeothermic heating blanket (Harvard Apparatus, Holliston, MA). A tracheostomy was performed to facilitate breathing. Supplementary oxygen was provided and if required, rats were artificially ventilated. The jugular and femoral veins were cannulated for the i.v. infusion of supplementary fluids (Ringer's solution 2.5 mL kg^−1^ h^−1^) and drugs, and the femoral artery cannulated for the recording of arterial pressure.

### Experimental protocol

After a 30 min stabilization period, each animal received graded i.v. doses of either sodium nitroprusside (SNP; 2.5, 5, 10, 20 and 40 *μ*g/kg) or adenosine (3, 10, 30, 100 and 300 *μ*g/kg), followed 1 h later by the alternate vasodilator. One hour after the final dose of SNP or adenosine was administered, each animal received cumulative doses of the ganglionic blocker hexamethonium (0.5, 1, 2, 4, 8, 20 and 40 mg/kg). At least 5 min separated the administration of each dose, and care was taken to ensure that all baseline cardiovascular variables were constant throughout the recording period (see Table 3). All the drugs were dissolved in saline and administered in boluses of 200 *μ*L over 10 sec to avoid fluid overload.

In a separate cohort of Lewis rats (*n* = 3), splanchnic SNA (*μ*V) was recorded as described previously (Harrison et al. 2010) and changes in SNA recorded in response to the graded i.v. boluses of hexamethonium. All SNA recordings were calibrated to a 50 *μ*V setting on the bioamplifier and made using the same electrode.

At the end of the experiment, animals were euthanized with an overdose of sodium pentobarbital (60 mg/kg by rapid i.v. injection, Virbac, Penrith, NSW, Australia). In animals in which splanchnic SNA was recorded, levels of SNA following euthanasia were recorded and subtracted from the original SNA trace.

### Data analysis

All data were acquired using a CED1401 (Cambridge Electronic Design Ltd., Cambridge, UK) and analyzed offline using Spike 2 (version 7; Cambridge Electronic Design Ltd.). Mean arterial pressure (MAP), diastolic blood pressure (DBP), pulse pressure (PP), and HR were derived from the arterial pressure signal. For determination of resting levels, arterial pressure was measured over a 1 min period prior to initiation of the experiment. For the experimental protocol, administration of each dose of SNP and adenosine were calculated as the peak change relative to a 30 sec period taken immediately prior to administration of each dose. As hexamethonium produced sustained responses, peak changes in SBP, MAP, DBP, PP, and HR in response to each dose of hexamethonium were calculated by taking a 1 min average at the nadir response, which was achieved within 2 min following each injection, and expressing this as change relative to the 30 sec period immediately prior to administration of the first dose of hexamethonium. All strains reached nadir response within this time frame, independent of the hexamethonium dose used.

Following the method of Moretti et al. 2009, in order to estimate the contribution of the vasculature to the magnitude of the depressor response to ganglionic blockade, the hypotensive response to hexamethonium was normalized with that of the vasodilators SNP and adenosine. The response to a single dose of hexamethonium was selected, being that dose after which no further fall in blood pressure was seen in all three strains (threshold). The dose of SNP and adenosine chosen for normalization was the highest dose at which the magnitude of the respective depressor response in the Lewis control strain matched that of the depressor response to 8 mg/kg hexamethonium. Using the doses of SNP and adenosine thus identified in the Lewis, the depressor response to 8 mg/kg hexamethonium was then normalized in all three strains. In contrast to the work of (Moretti et al. 2009), our data were analyzed for not only MAP responses but also for SBP in order to account for the widened PP observed in the LPK rats. We further used only a single dose of SNP for our calculations, rather than the average of multiple doses, and the normalized responses to SNP and adenosine were analyzed separately.

In order to examine the relationship between the LF power of SBPV and SBP during ganglionic blockade (Diedrich et al. 2003), an 80 sec period immediately prior to administration of the first dose of hexamethonium and at nadir following administration of each hexamethonium dose (0.5, 1, 2, 4 and 8 mg/kg) was taken. For each 80 sec segment analyzed, the average SBP was calculated. Normalized low frequency power (LF_nu_) was then calculated by expressing the absolute LF power as a ratio of the combined low and high frequency power for each 80 sec segment as described previously (Hildreth et al. 2013). The amount of LF_nu_ power and the corresponding SBP value was determined and linear regression analysis used to calculate the slope of this relationship within each strain and this value compared between the strains.

### Statistical analysis

Values are expressed as mean ± standard error of mean (SEM). All data sets were initially analyzed using two‐way ANOVA with sex as a covariate. This analysis indicated no sex effect (*P *>**0.05 all data sets) and therefore data from both male and female animals were combined. A one‐way ANOVA with Bonferroni's corrections was used to identify differences in the blood pressure and HR responses and the ratio of MAP and SBP response of hexamethonium/SNP and hexamethonium/adenosine between the LPK and Lewis or SHR. A two‐way ANOVA with Bonferroni's corrections with strain and dose as variables was used to identify dose‐dependent responses in the MAP and SBP response to SNP, adenosine and hexamethonium between the three strains including threshold response. A Bartlett's test was used to determine if there were any differences in the variance, and if so, the data were log‐transformed before statistical analysis. The relationship between SBP and LF_nu_ power of SBPV was analyzed using linear regression. All statistical analyses were performed using GraphPad Prism (v.6, GraphPad Software Inc., La Jolla, CA). Significance was defined as *P *≤**0.05.

## Results

### Resting cardiovascular parameters

Resting measurements of SBP, MAP, DBP, PP, and HR are summarized in Table 1. The LPK exhibited elevated SBP and MAP compared with Lewis rats, but not SHR, while DBP was lower compared with SHR but not Lewis. Pulse pressure was larger in the LPK compared with both Lewis rats and SHR. Resting HR did not differ between strains.

**Table 1.** Resting blood pressure and heart rate levels in the Lewis, Lewis polycystic kidney (LPK) and spontaneously hypertensive rat (SHR)

Parameter	Lewis *n = *13	LPK *n = *12	SHR *n = *12
SBP (mmHg)	130 ± 2	203 ± 9^*^^,^^#^	174 ± 7
MAP (mmHg)	84 ± 2	113 ± 6^*^	117 ± 4
DBP (mmHg)	61 ± 3	70 ± 6^#^	88 ± 6
PP (mmHg)	69 ± 3	133 ± 10^*^^,^^#^	85 ± 10
HR (BPM)	362 ± 13	360 ± 15	335 ± 10

SBP, systolic blood pressure; MAP, mean arterial pressure; DBP, diastolic blood pressure; PP, pulse pressure; HR, heart rate; and BPM, beats per minute. Results are expressed as mean ± SEM.

*P <**0.01 compared to Lewis and ^#^*P *<**0.01 compared to SHR.

### Cardiovascular responses to administration of direct acting vasodilators

**Figure 1.**
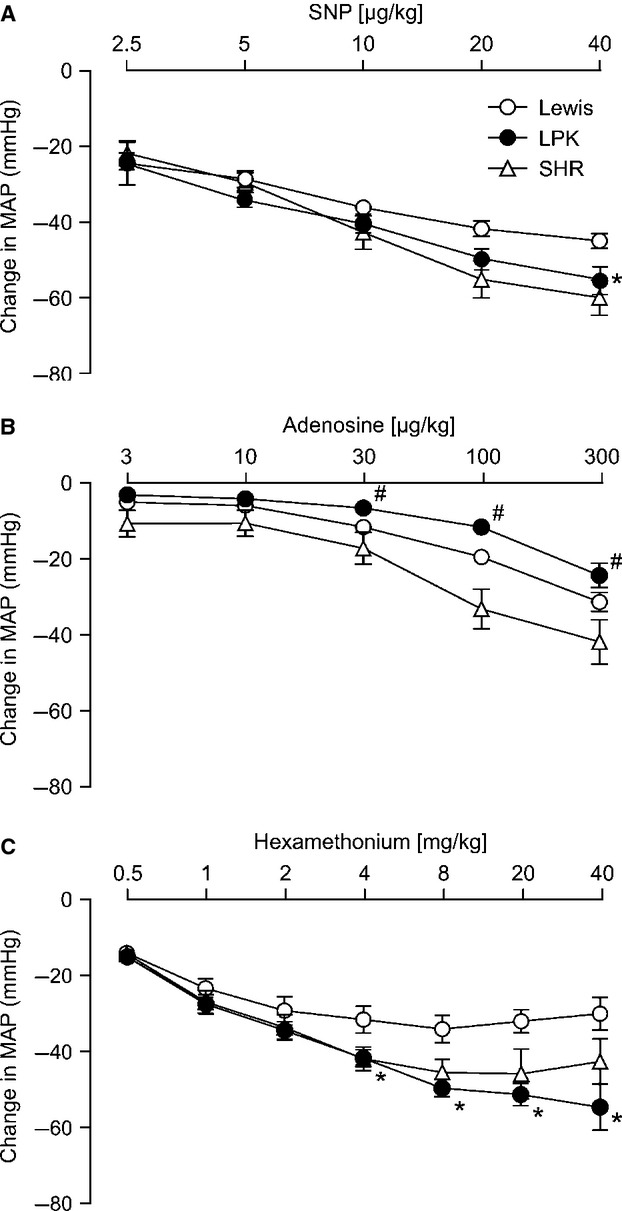
Dose dependent effects of sodium nitroprusside (SNP; A), adenosine (B) and the ganglionic blocker hexamethonium (C) on mean arterial pressure (MAP) in Lewis, Lewis polycystic kidney (LPK) and spontaneously hypertensive rats (SHR). Results are expressed as mean ± SEM. *P <**0.05 compared to Lewis and ^#^*P *<**0.05 compared to SHR for individual doses as indicated.

**Figure 2.**
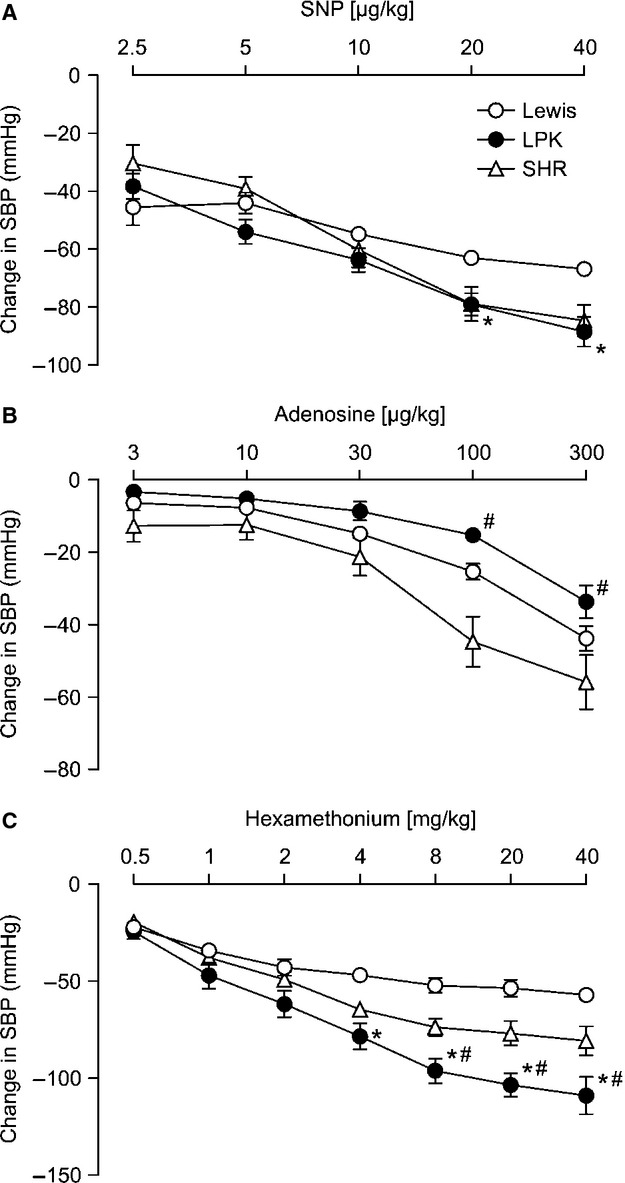
Dose dependent effects of sodium nitroprusside (SNP; A), adenosine (B) and the ganglionic blocker hexamethonium (C) on systolic blood pressure (SBP) in Lewis, Lewis polycystic kidney (LPK) and spontaneously hypertensive rats (SHR). Results are expressed as mean ± SEM. *P <**0.05 compared to Lewis and ^#^*P *<**0.05 compared to SHR for individual doses as indicated.

In response to administration of SNP, the two‐way ANOVA identified both a dose‐ and strain‐dependent effect on MAP and SBP (*P *<**0.001). This was evident as a dose‐dependent reduction in all strains (Figs. 1A, 2A). In the Lewis, maximal depressor responses to SNP were observed following 10 *μ*g/kg for both MAP and SBP; whereas, in the LPK and SHR, maximal depressor responses to SNP (MAP and SBP) were seen with 20 *μ*g/kg. The overall response in the LPK was significantly greater than that in the Lewis for both MAP and SBP. No differences were identified between LPK and SHR at any dose of SNP. Hemodynamic responses to SNP at 20 *μ*g/kg are presented in Table 2. At this dose, there was also a significant difference in the PP response between the LPK versus Lewis rats. Absolute values are provided in Table 3.

**Table 2.** Average changes in blood pressure and heart rate in response to direct vasodilators and ganglionic blockade in the Lewis, Lewis polycystic kidney (LPK), and spontaneously hypertensive rat (SHR)

Drug	Parameter	Lewis *n = *8	LPK *n *=**9	SHR *n = *11
SNP (20 *μ*g/kg)	SBP	−63 ± 2	−79 ± 4^*^	−79 ± 6
	MAP	−43 ± 2	−50 ± 3	−55 ± 5
	DBP	−33 ± 2	−35 ± 3	−43 ± 5
	PP	−30 ± 2	−44 ± 4^*^	−36 ± 3
	HR	12 ± 3	18 ± 4	11 ± 2
Adenosine (300 *μ*g/kg)	SBP	−44 ± 3	−34 ± 4^#^	−56 ± 7
	MAP	−32 ± 3	−24 ± 3^#^	−42 ± 6
	DBP	−26 ± 3	−20 ± 3^#^	−35 ± 5
	PP	−18 ± 2	−14 ± 3	−21 ± 4
	HR	−8 ± 4	−1 ± 6^#^	−64 ± 14
Hexamethonium (8 mg/kg)	SBP	−52 ± 4	−96 ± 6^*^^,^^#^	−74 ± 4
	MAP	−31 ± 3	−51 ± 3^*^	−46 ± 3
	DBP	−20 ± 3	−28 ± 3	−32 ± 4
	PP	−32 ± 4	−69 ± 7^*^^,^^#^	−42 ± 5
	HR	−19 ± 6	−2 ± 18	−10 ± 7

All blood pressure responses are expressed as mmHg. Heart rate is expressed as beats per minute. SBP, systolic blood pressure; MAP, mean arterial pressure; DBP, diastolic blood pressure; PP, pulse pressure; HR, heart rate. Results are expressed as mean ± SEM.

*P <**0.05 compared to Lewis and ^#^*P *<**0.05 compared to SHR. *n* represents minimum number per group.

**Table 3.** Average blood pressure and heart rate values before (baseline) and in response to direct vasodilators and ganglionic blockade in the Lewis, Lewis polycystic kidney (LPK), and spontaneously hypertensive rat (SHR)

Strain	Parameter	Baseline	SNP 20 *μ*g/kg	Baseline	Adenosine 300 *μ*g/kg	Baseline	Hex 8 mg/kg
Lewis (*n *=**8)	SBP	133 ± 3	70 ± 2	129 ± 4	84 ± 3	125 ± 3	73 ± 3
	MAP	89 ± 2	46 ± 2	84 ± 3	52 ± 2	82 ± 3	52 ± 3
	DBP	67 ± 3	34 ± 2	62 ± 3	35 ± 1	61 ± 4	41 ± 3
	PP	66 ± 3	36 ± 3	67 ± 3	49 ± 2	65 ± 4	32 ± 1
	HR	366 ± 12	377 ± 13	369 ± 13	362 ± 14	376 ± 11	357 ± 12
LPK (*n *=**9)	SBP	206 ± 9	127 ± 8	202 ± 9	168 ± 8	212 ± 9	115 ± 8
	MAP	119 ± 5	69 ± 4	113 ± 5	89 ± 4	115 ± 6	65 ± 5
	DBP	75 ± 5	40 ± 3	70 ± 5	50 ± 4	67 ± 6	40 ± 4
	PP	130 ± 8	86 ± 7	132 ± 10	118 ± 8	144 ± 7	76 ± 5
	HR	351 ± 14	369 ± 15	359 ± 17	358 ± 15	351 ± 18	349 ± 19
SHR (*n *=**11)	SBP	176 ± 6	97 ± 4	170 ± 6	114 ± 6	169 ± 8	95 ± 6
	MAP	117 ± 5	62 ± 4	112 ± 3	70 ± 6	111 ± 3	65 ± 2
	DBP	87 ± 6	45 ± 5	83 ± 5	48 ± 8	82 ± 6	50 ± 3
	PP	89 ± 8	52 ± 6	87 ± 10	66 ± 9	87 ± 12	45 ± 8
	HR	338 ± 11	349 ± 12	329 ± 10	267 ± 18	336 ± 11	326 ± 8

All blood pressure responses are expressed as mmHg. Heart rate is expressed as beats per minute. SNP, sodium nitroprusside; Hex, Hexamethonium; SBP, systolic blood pressure; MAP, mean arterial pressure; DBP, diastolic blood pressure; PP, pulse pressure; HR, heart rate. Results are expressed as mean ± SEM. *n* represents minimum number per group.

In response to administration of adenosine, the two‐way ANOVA identified both a dose‐ and strain‐dependent effect on MAP and SBP (*P *<**0.001). This was evident as a dose‐dependent reduction in all strains (Figs. 1B, 2B). The MAP response in both the Lewis and SHR reached saturation at 100 *μ*g/kg adenosine, while in the LPK, the MAP depressor response did not reach saturation, with the depressor response to 300 *μ*g/kg adenosine being greater than that observed with 100 *μ*g/kg adenosine (*P *<**0.05). The SBP response did not reach saturation in either the Lewis or LPK, being greater at 300 *μ*g/kg adenosine than for 100 *μ*g/kg adenosine (*P *<**0.05). In the SHR, the threshold SBP depressor response to adenosine was observed at 100 *μ*g/kg, with no further reduction observed with 300 *μ*g/kg (*P *>**0.05). Overall, a greater depressor response was observed in the SHR compared with LPK in response to adenosine for both MAP and SBP (Figs. 1B, 2B). Hemodynamic responses to adenosine at 300 *μ*g/kg are presented in Table 2. Differences in response to 300 *μ*g/kg adenosine were also seen for DBP, being significantly less in LPK versus SHR however changes in PP were comparable between the strains. There was a marked bradycardia observed in the SHR in response to adenosine that was not apparent in the LPK or Lewis (Table 2). Absolute values are provided in Table 3.

### Cardiovascular responses to administration of the ganglionic blocker hexamethonium

In response to administration of hexamethonium, the two‐way ANOVA identified both a dose and strain dependent effect on MAP and SBP (*P *<**0.001). This was evident as a dose‐dependent reduction in all strains (Figs. 1C, 2C). In the Lewis, maximal reduction in MAP and SBP was achieved using 2 mg/kg hexamethonium, with no further change observed with the higher doses. In the LPK, maximal reduction in MAP and SBP was seen at 8 mg/kg hexamethonium; while in the SHR, saturation in MAP and SBP responses were evident at 4 mg/kg hexamethonium. Hemodynamic responses to 8 mg/kg hexamethonium, being the single dose to evoke maximal changes in blood pressure in all three strains, are provided in (Table 2). There was no significant difference in the DBP response between the strains, however, the change in PP in the LPK was significantly greater than that for either the Lewis controls or SHR. There was no significant difference in the change in HR between the three strains. Absolute values are provided in Table 3. After 8 mg/kg hexamethonium, the absolute MAP and SBP level was still greater in the LPK than that seen in Lewis controls (*P *≤**0.05) and PP in the LPK rats was higher relative to both Lewis rats and SHR (*P *<**0.001).

In order to confirm that 8 mg/kg hexamethonium produced maximal cessation of sympathetic outflow, splanchnic SNA was recorded in a separate group of Lewis rats (*n* = 3). Baseline splanchnic SNA was significantly reduced following administration of hexamethonium (3.7 ± 0.9 vs. 0.24 ± 0.09 *μ*V, baseline vs. 8 mg/kg hexamethonium *P *<**0.001). Subsequent administration of higher doses of hexamethonium produced no further reduction in SNA (8 vs. 20 vs. 40 mg/kg hexamethonium *P *>**0.99).

### Normalization of the response to ganglionic blockade

In order to identify if a vascular amplifier effect was responsible for the enhanced depressor responses to hexamethonium in the LPK, the ratio of the depressor response relative to the direct vasodilators was calculated. The rationale being that when the ratio of these responses was matched in the control animals, if the ratio to the same dose of the respective drugs in the LPK rats was comparable, it would indicate a vascular amplifier effect underlies the enhanced depressor response to hexamethonium observed in this strain, whereas if the ratio was greater, then the depressor response to hexamethonium reflects heightened sympathetic tone.

**Figure 3.**
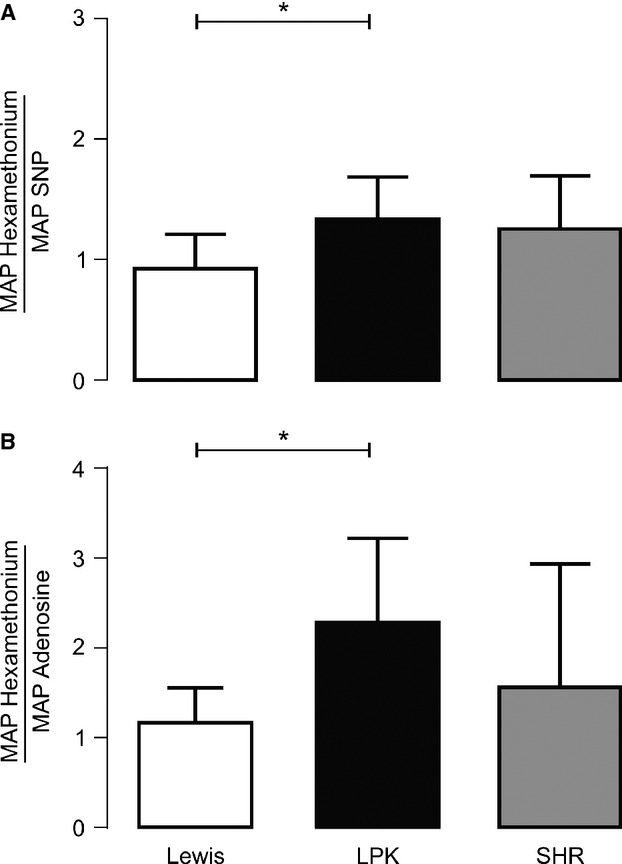
Ratio of the mean arterial pressure (MAP) response after ganglionic blockade (hexamethonium 8 mg/kg i.v.) to that of the direct acting vasodilators (A: sodium nitroprusside [SNP 10 *μ*g/kg i.v.] and B: adenosine [300 *μ*g/kg i.v.]) in Lewis, Lewis polycystic kidney (LPK) and spontaneously hypertensive rats (SHR). Results are expressed as mean ± SEM. *P <**0.05 compared to Lewis.

**Figure 4.**
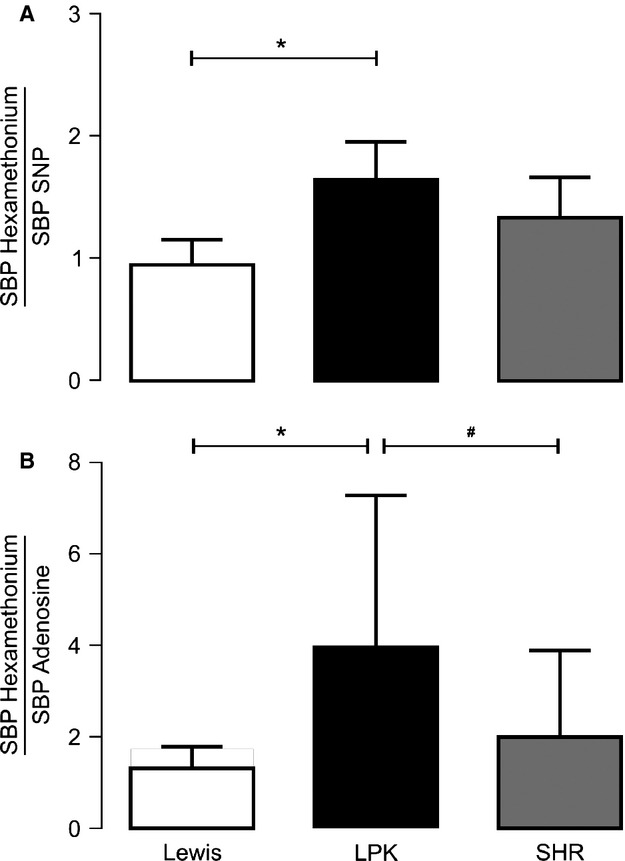
Ratio of the systolic blood pressure (SBP) response after ganglionic blockade (hexamethonium 8 mg/kg i.v.) to that of the direct acting vasodilators (A: sodium nitroprusside [SNP 10 *μ*g/kg i.v.] and B: adenosine [300 *μ*g/kg i.v.]) in Lewis, Lewis polycystic kidney (LPK) and spontaneously hypertensive rats (SHR). Results are expressed as mean ± SEM. *P <**0.05 compared to Lewis and ^#^*P *<**0.05 compared to SHR.

The response to 8 mg/kg hexamethonium was used for normalization as it was the dose at which no further fall in either MAP or SBP was seen across all three strains. In Lewis control rats, the doses of SNP and adenosine that produced comparable reductions in MAP and SBP to 8 mg/kg hexamethonium were identified. For SNP, this was identified as 10 *μ*g/kg for both MAP and SBP (MAP: hexamethonium 8 mg/kg −35 ± 4 vs. SNP 10 μg/kg −36 ± 1 mmHg; SBP: hexamethonium 8 mg/kg −52 ± 4 vs. SNP 10 μg/kg: −55 ± 2 mmHg, both *P *>**0.05). For adenosine it was determined to be 300 *μ*g/kg for both MAP and SBP (MAP: hexamethonium 8 mg/kg −35 ± 4 vs. adenosine 300 *μ*g/kg −31 ± 2 mmHg; SBP: hexamethonium 8 mg/kg: −52 ± 4 vs. adenosine 300 μg/kg −45 ± 3 mmHg; both *P *>**0.05). Consequently, the depressor responses to 10 *μ*g/kg SNP and 300 *μ*g/kg adenosine were used to normalize the response to ganglionic blockade (both MAP and SBP) in all three strains. The ratio of the reduction in MAP in response to administration of hexamethonium/SNP and hexamethonium/adenosine is illustrated in Fig. 3. In the LPK, both of these ratios were greater than that obtained in the Lewis controls. No difference in these ratios relative to the SHR was observed in the LPK. When these ratios were calculated using SBP (Fig. 4), both ratios were again greater in the LPK compared with Lewis and the adenosine response was also significantly greater than that of the SHR.

### Relationship between LF power and SBP following ganglionic blockade

In order to identify if LF power was reflective of sympathetic tone, the slope of the relationship between LF_nu_ power and SBP in the presence of ganglionic blockade was compared. If LF power was reflective of sympathetic tone, then the slope of the relationship would be greater compared with the Lewis rat. If not, then the slope would be comparable to that observed in the Lewis.

**Figure 5.**
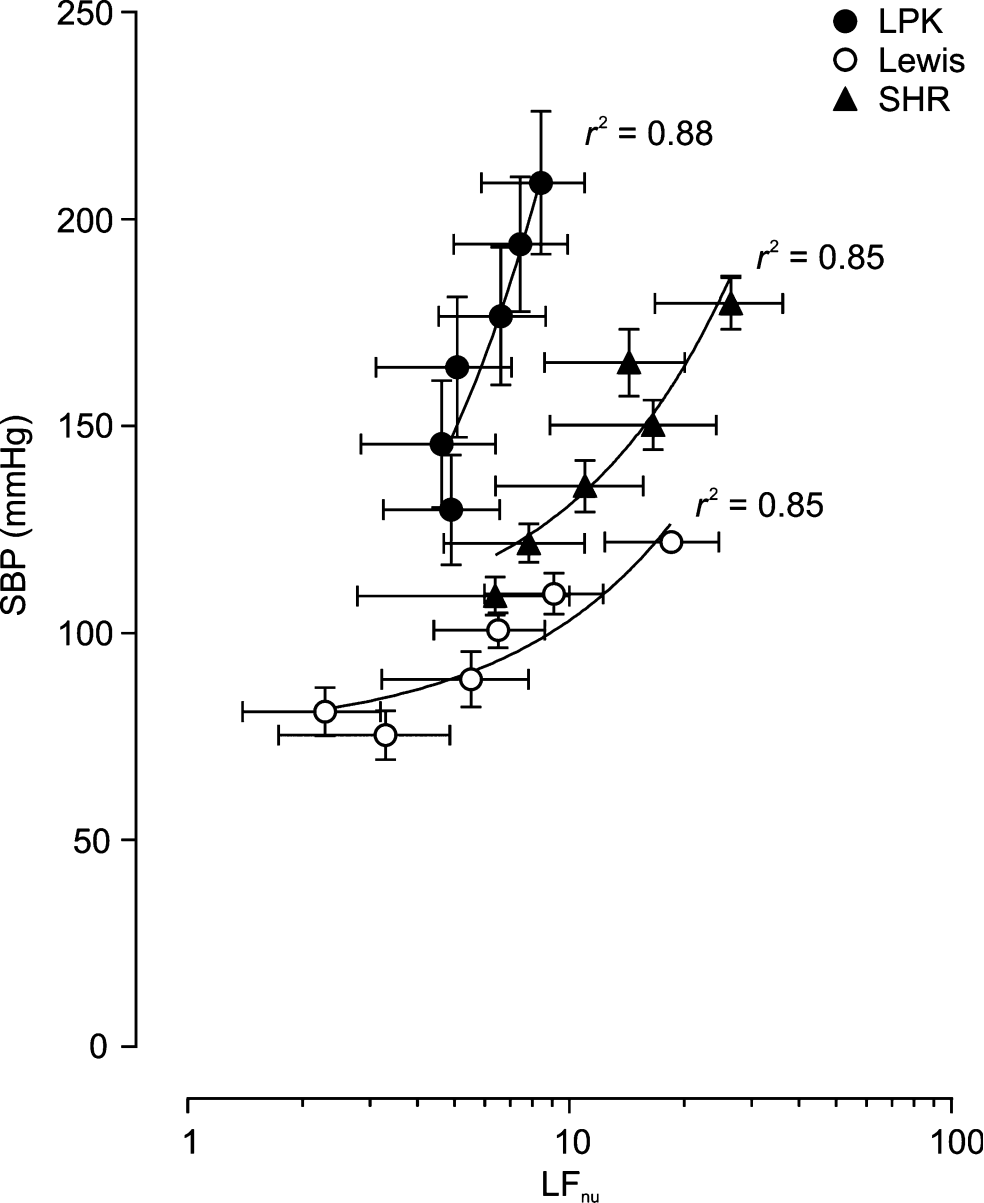
Systolic blood pressure (SBP) plotted against the normalized unit of the low frequency SBP power LF_nu_ during administration of graded i.v. boluses of the ganglionic blocker hexamethonium in Lewis, Lewis polycystic kidney (LPK) and spontaneously hypertensive rats (SHR). The relationship between SBP and LF_nu_ power of SBP variability was analyzed using linear regression. The regression *r*^2^ values are as detailed in the Figure.

The relationship between the LF_nu_ power and SBP in the presence of ganglionic blockade is shown in Fig. 5. The slope was greater in the LPK compared with both Lewis (17.93 ± 3.26 vs. 2.78 ± 0.59 mmHg/LF_nu_, respectively, *P *=**0.001) and SHR (3.36 ± 0.72 mmHg/LF_nu_, *P *=**0.003).

## Discussion

In the present study, we sought to identify if a vascular amplifier was contributing to the heightened depressor responses to ganglionic blockade and the increased LF oscillations in SBP that we have previously reported in the hypertensive LPK model of CKD (Phillips et al. 2007; Harrison et al. 2010; Hildreth et al. 2013). Our findings indicate that sympathetic overactivity prevails over any presence of a vascular amplifier in this model.

In the LPK rats, the normalized depressor response to ganglionic blockade was greater than that seen in the Lewis controls for either of the direct acting vasodilators: SNP or adenosine, indicating that in this model of CKD, the enhanced depressor response is reflective of increased sympathetic vasomotor tone. This was evident when responses were assessed for both MAP and SBP. Our data, however, do not rule out a role of the vasculature in contributing to the maintained hypertension in this model, highlighted in particular by the finding that following administration of hexamethonium, blood pressure remained elevated relative to Lewis. Previously, we demonstrated that the LPK has increased pulse wave velocity, a functional index of arterial stiffness, as well as large vessel remodeling (Ng et al. 2011), and it is likely that vascular changes contribute to the additional, nonsympathetic level of increased blood pressure observed in the LPK. Direct assessment of resistance vessels, examining both structural characteristics and functional responses including those mediated by adrenergic receptors, will be required to further delineate the contribution of the vasculature.

An additional robust means by which to examine the contribution of the SNS to the maintenance of blood pressure is the slope of the relationship between LF oscillation in SBP and resting SBP under ganglionic blockade (Diedrich et al. 2003; Head 2003), with a greater slope indicative of increased sympathetic vasomotor tone. In accordance with our other findings, the slope of the relationship between LF power and SBP was greater in the LPK when compared to Lewis. This suggests that the increased LF power we have reported in this model previously (Harrison et al. 2010) is reflective of increased sympathetic vasomotor tone. In the SHR, however, the slope of this relationship was reduced in comparison with the LPK. This is consistent with previous studies (Stauss et al. 1995) showing that despite an increase in splanchnic SNA in the SHR compared to normotensive Wistar Kyoto control rats, the coherence between SNA and LF power was not greater and could not be reduced by *α*_1_‐adrenoceptor blockade using prazosin. Together, this suggests that under basal conditions, LF power does not reflect sympathetic vasomotor tone in the SHR and that it may be driven by non‐neurogenic vascular properties.

Across all three strains DBP changed similarly in response to administration of hexamethonium and it was the systolic component that drove the difference in MAP. This is an important consideration given the significance of systolic over diastolic pressure as a risk marker for cardiovascular disease (Franklin et al. 1999; London and Guerin 1999). Another notable feature of our data was the widened PP in the LPK rats and that it was reduced to a greater extent by hexamethonium than in either SHR or Lewis animals. When assessing the data as MAP therefore, these factors combined (i.e., widened PP and greater SBP responses) could mask potentially significant effects when considering the normalized ganglionic blockade response. Notwithstanding therefore the contribution of arterial compliance and stroke volume to SBP (London and Guerin 1999), it was important to analyze both MAP and SBP to verify the existence or otherwise of a vascular amplifier. Importantly, we show that sympathetic overactivity prevails over any presence of a vascular amplifier in the LPK regardless of whether the data were analyzed using MAP or SBP.

A number of methodological issues need to be considered in the interpretation of our results, including the use of an anesthetized preparation, the contribution of cardiac output and the role of counter‐regulatory blood pressure mechanisms. Urethane anesthesia has been shown to both promote and inhibit SNA (Shimokawa et al. 1998; Wang et al. 2014), and a potential confounding effect therefore is altered baseline levels of blood pressure due to the anesthetic. It is also possible that anesthesia may influence the response to pharmacological testing. For example, the MAP response to hexamethonium has been shown to be different in anesthetized versus conscious animals, in both normotensive and hypertensive models (Biancardi et al. 2007). Future studies examining both SNA and blood pressure in conscious animals are required to address this issue.

Secondly, we did not pace the heart and therefore the blood pressure changes we observed may be contributed to by changes in cardiac output and not vasodilation alone. This may not be an issue with respect to hexamethonium, as at least under conscious conditions, the depressor response is believed to be mediated by vasodilation alone (Fink and Ploucha 1986; Osborn et al. 1987; Santajuliana et al. 1996). Furthermore, we saw comparable HR changes in response to hexamethonium in the LPK compared with both Lewis and SHR, indicating that at least the HR changes produced by the concomitant cardiac autonomic blockade are not contributing to the depressor responses we observed. Nevertheless, this is an important consideration for the depressor responses to administration of direct vasodilators and certainly with respect to the depressor responses obtained following adenosine. In the SHR, adenosine produced a marked bradycardia that was not present in either the LPK or Lewis. It is therefore highly probable that the resultant change in cardiac output contributed to the marked depressor response observed in the SHR and calls into question the use of adenosine as a direct acting vasodilator in which to assess the role of a vascular amplifier.

Finally, the blood pressure changes observed may be influenced by activation of the baroreflex (in response to direct acting vasodilators) or by the release of vasoactive hormones (in response to hexamethonium). With respect to the role of the baroreflex, while we did not control for this, we have previously shown the baroreflex is impaired in the LPK (Harrison et al. 2010; Hildreth et al. 2013; Salman et al. 2014). As such, it would be expected that the reduced ability to buffer acute changes in blood pressure would result in larger depressor responses. While we did see greater depressor responses to SNP, we did not observe heighted depressor responses to adenosine. Therefore, the fact that we observe a greater ratio when normalized with either vasodilator supports our assertion that increased sympathetic tone prevails over a vascular amplifier. With respect to the role of hormonal changes, it is possible that hexamethonium produced counter‐regulatory increases in angiotensin II or vasopressin. While it has been shown previously that depressor responses to hexamethonium in conscious Sprague Dawley rats are not influenced by prior blockade of either angiotensin II or vasopressin receptors (Santajuliana et al. 1996), it is possible that these mechanisms contribute under anesthesia. Notwithstanding these limitations, the approaches used in this paper, as of those used by Moretti (Moretti et al. 2009) and Diedrich (Diedrich et al. 2003), provide a valuable means by which researchers can better interpret responses to ganglionic blockade as an indicator of sympathetic vasomotor tone.

## Conclusion

In the present study, we demonstrate that in the LPK model of CKD, depressor responses to ganglionic blockade as well as enhanced LF oscillations in SBP reflect increased sympathetic vasomotor tone, as opposed to a vascular driven amplification of vasoactive input. This supports our hypothesis that sympathetic overactivity is a significant contributor to the maintenance of hypertension in this rodent model.

## Acknowledgements

The authors thank Dr Ibrahim M. Salman, from the Australian School of Advanced Medicine, Macquarie University, for his assistance in the nerve recording experiments.

## Conflict of interest

The authors have no conflicts of interest to declare.

## References

[b1] Abdala A. P. McBryde F. D. Marina N. Hendy E. B. Engelman Z. J. Fudim M. 2012 Hypertension is critically dependent on the carotid body input in the spontaneously hypertensive rat. J. Physiol.; 590:4269-4277. 22687617 10.1113/jphysiol.2012.237800 PMC3473284

[b2] Adams M. A. Bobik A. Korner P. I. 1989 Differential development of vascular and cardiac hypertrophy in genetic hypertension. Relation to sympathetic function. Hypertension; 14:191-202. 2527201 10.1161/01.hyp.14.2.191

[b3] Adams M. A. Bobik A. Korner P. I. 1990 Enalapril can prevent vascular amplifier development in spontaneously hypertensive rats. Hypertension; 16:252-260. 2394485 10.1161/01.hyp.16.3.252

[b4] Bevan R. D. 1984 Trophic effects of peripheral adrenergic nerves on vascular structure. Hypertension; 6:III19-III26. 6394491 10.1161/01.hyp.6.6_pt_2.iii19

[b5] Biancardi V. C. Bergamaschi C. T. Lopes O. U. Campos R. R. 2007 Sympathetic activation in rats with L‐NAME‐induced hypertension. Braz. J. Med. Biol. Res.; 40:401-408. 17334538

[b6] Black M. J. Kanellakis P. Bobik A. 1997 Role of angiotensin II in early cardiovascular growth and vascular amplifier development in spontaneously hypertensive rats. J. Hypertens.; 15:945-954. 9321741 10.1097/00004872-199715090-00004

[b7] Diedrich A. Jordan J. Tank J. Shannon J. R. Robertson R. Luft F. C. 2003 The sympathetic nervous system in hypertension: assessment by blood pressure variability and ganglionic blockade. J. Hypertens.; 21:1677-1686. 12923400 10.1097/00004872-200309000-00017

[b8] Fink G. D. Ploucha J. M. 1986 Contribution of the autonomic nervous system and vasopressin to elevated vascular resistance in the spontaneously hypertensive rat. J. Hypertens.; 4Suppl. 6:S594-S596.

[b9] Folkow B. Karlstrom G. 1984 Age‐ and pressure‐dependent changes of systemic resistance vessels concerning the relationships between geometric design, wall distensibility, vascular reactivity and smooth muscle sensitivity. Acta Physiol. Scand.; 122:17-33. 6507119 10.1111/j.1748-1716.1984.tb07477.x

[b10] Franklin S. S. Khan S. A. Wong N. D. Larson M. G. Levy D. 1999 Is pulse pressure useful in predicting risk for coronary heart Disease? The Framingham heart study. Circulation; 100:354-360. 10421594 10.1161/01.cir.100.4.354

[b11] Grassi G. Quarti‐Trevano F. Seravalle G. Arenare F. Volpe M. Furiani S. 2011 Early sympathetic activation in the initial clinical stages of chronic renal failure. Hypertension; 57:846-851. 21300663 10.1161/HYPERTENSIONAHA.110.164780

[b12] Harrison J. L. Hildreth C. M. Callahan S. M. Goodchild A. K. Phillips J. K. 2010 Cardiovascular autonomic dysfunction in a novel rodent model of polycystic kidney disease. Auton. Neurosci.; 152:60-66. 19825515 10.1016/j.autneu.2009.09.019

[b13] Head G. A. 2003 The sympathetic nervous system in hypertension: assessment by blood pressure variability and ganglionic blockade. J. Hypertens.; 21:1619-1621. 12923389 10.1097/00004872-200309000-00006

[b14] Hildreth C. M. Kandukuri D. S. Goodchild A. K. Phillips J. K. 2013 Temporal development of baroreceptor dysfunction in a rodent model of chronic kidney disease. Clin. Exp. Pharmacol. Physiol.; 40:458-465. 23662737 10.1111/1440-1681.12110

[b15] Johansson M. Elam M. Rundqvist B. Eisenhofer G. Herlitz H. Lambert G. 1999 Increased sympathetic nerve activity in renovascular hypertension. Circulation; 99:2537-2542. 10330385 10.1161/01.cir.99.19.2537

[b16] Judy W. V. Watanabe A. M. Henry D. P. Besch H. R. Jr Murphy W. R. Hockel G. M. 1976 Sympathetic nerve activity: role in regulation of blood pressure in the spontaenously hypertensive rat. Circ. Res.; 38:21-29. 178466 10.1161/01.res.38.6.21

[b17] Klein I. H. Ligtenberg G. Oey P. L. Koomans H. A. Blankestijn P. J. 2001 Sympathetic activity is increased in polycystic kidney disease and is associated with hypertension. J. Am. Soc. Nephrol.; 12:2427-2433. 11675419 10.1681/ASN.V12112427

[b18] Li Q. Dale W. E. Hasser E. M. Blaine E. H. 1996 Acute and chronic angiotensin hypertension: neural and nonneural components, time course, and dose dependency. Am. J. Physiol.; 271:R200-R207. 8760221 10.1152/ajpregu.1996.271.1.R200

[b19] London G. M. Guerin A. 1999 Influence of arterial pulse and reflective waves on systolic blood pressure and cardiac function. J. Hypertens. Suppl.; 17:S3-S6. 10465060

[b20] McCooke J. K. Appels R. Barrero R. A. Ding A. Ozimek‐Kulik J. E. Bellgard M. I. 2012 A novel mutation causing nephronophthisis in the Lewis polycystic kidney rat localises to a conserved RCC1 domain in Nek8. BMC Genom.; 13:1471-2164. 10.1186/1471-2164-13-393 PMC3441220 22899815

[b21] Moretti J. L. Burke S. L. Evans R. G. Lambert G. W. Head G. A. 2009 Enhanced responses to ganglion blockade do not reflect sympathetic nervous system contribution to angiotensin II‐induced hypertension. J. Hypertens.; 27:1838-1848. 19512943 10.1097/HJH.0b013e32832dd0d8

[b22] Murphy C. A. Sloan R. P. Myers M. M. 1991 Pharmacologic responses and spectral analyses of spontaneous fluctuations in heart rate and blood pressure in SHR rats. J. Auton. Nerv. Syst.; 36:237-250. 1787260 10.1016/0165-1838(91)90047-7

[b23] Ng K. Hildreth C. M. Phillips J. K. Avolio A. P. 2011 Aortic stiffness is associated with vascular calcification and remodeling in a chronic kidney disease rat model. Am. J. Physiol. Renal. Physiol.; 300:F1431-F1436. 21478483 10.1152/ajprenal.00079.2011

[b24] Osborn J. W. Jr Skelton M. M. Cowley A. W. Jr 1987 Hemodynamic effects of vasopressin compared with angiotensin II in conscious rats. Am. J. Physiol.; 252:H628-H637. 3826404 10.1152/ajpheart.1987.252.3.H628

[b25] Phillips J. K. Hopwood D. Loxley R. A. Ghatora K. Coombes J. D. Tan Y. S. 2007 Temporal relationship between renal cyst development, hypertension and cardiac hypertrophy in a new rat model of autosomal recessive polycystic kidney disease. Kidney Blood Press. Res.; 30:129-144. 17446713 10.1159/000101828

[b26] Salman I. M. Hildreth C. M. Ameer O. Z. Phillips J. K. 2014 Differential contribution of afferent and central pathways to the development of baroreflex dysfunction in chronic kidney disease. Hypertension; 63:804-810. 24379179 10.1161/HYPERTENSIONAHA.113.02110

[b27] Santajuliana D. Hornfeldt B. J. Osborn J. W. 1996 Use of ganglionic blockers to assess neurogenic pressor activity in conscious rats. J. Pharmacol. Toxicol. Methods; 35:45-54. 8645881 10.1016/1056-8719(95)00132-8

[b28] Shannon J. R. Flattem N. L. Jordan J. Jacob G. Black B. K. Biaggioni I. 2000 Orthostatic intolerance and tachycardia associated with norepinephrine‐transporter deficiency. N. Engl. J. Med.; 342:541-549. 10684912 10.1056/NEJM200002243420803

[b29] Shimokawa A. Kunitake T. Takasaki M. Kannan H. 1998 Differential effects of anesthetics on sympathetic nerve activity and arterial baroreceptor reflex in chronically instrumented rats. J. Auton. Nerv. Syst.; 72:46-54. 9760079 10.1016/s0165-1838(98)00084-8

[b30] Smid S. D. Frewin D. B. Wyartt C. L. Head R. J. 1995 Functional tolerance to alpha‐adrenergic receptor blockade in the spontaneously hypertensive rat highlights the multifunctional role of vascular angiotensin II in the development of hypertension. J. Vasc. Res.; 32:247-253. 7654881 10.1159/000159099

[b31] Stauss H. M. 2007 Identification of blood pressure control mechanisms by power spectral analysis. Clin. Exp. Pharmacol. Physiol.; 34:362-368. 17324151 10.1111/j.1440-1681.2007.04588.x

[b32] Stauss H. M. Mrowka R. Nafz B. Patzak A. Unger T. Persson P. B. 1995 Does low frequency power of arterial blood pressure reflect sympathetic tone? J. Auton. Nerv. Syst.; 54:145-154. 7499726 10.1016/0165-1838(94)00000-a

[b33] Vanness J. M. Hinojosa‐Laborde C. Craig T. Haywood J. R. 1999 Effect of sinoaortic deafferentation on renal wrap hypertension. Hypertension; 33:476-481. 9931151 10.1161/01.hyp.33.1.476

[b34] Wang G. F. Mao X. J. Chen Z. J. 2014 Urethane suppresses renal sympathetic nerve activity in Wistar rats. Eur. Rev. Med. Pharmacol. Sci.; 18:1454-1457. 24899602

[b35] Wright C. E. Angus J. A. 1999 Enhanced total peripheral vascular responsiveness in hypertension accords with the amplifier hypothesis. J. Hypertens.; 17:1687-1696. 10658934 10.1097/00004872-199917120-00005

[b36] Zoccali C. Mallamaci F. Parlongo S. Cutrupi S. Benedetto F. A. Tripepi G. 2002 Plasma norepinephrine predicts survival and incident cardiovascular events in patients with end‐stage renal disease. Circulation; 105:1354-1359. 11901048 10.1161/hc1102.105261

